# Tumor Necrosis on Routine Pretreatment CT as a Potential Prognostic Marker in Advanced NSCLC Treated With Immune Checkpoint Inhibitor Monotherapy: A Two‐Center Retrospective Study

**DOI:** 10.1111/1759-7714.70363

**Published:** 2026-07-26

**Authors:** Eiji Takeuchi, Hirokazu Ogino, Kensuke Kondo, Yoshio Okano, Hiroki Takahashi, Kaori Nii, Yugo Matsumura, Takuya Ishida, Seiya Ichihara, Michihiro Kunishige, Naoki Kadota, Hisanori Machida, Nobuo Hatakeyama, Keishi Naruse, Seidai Sato, Hiroshi Nokihara, Tsutomu Shinohara, Yasuhiko Nishioka

**Affiliations:** ^1^ Department of Clinical Investigation National Hospital Organization Kochi Hospital Kochi Japan; ^2^ Department of Respiratory Medicine and Rheumatology Graduate School of Biomedical Sciences, Tokushima University Tokushima Japan; ^3^ Department of Respiratory Medicine National Hospital Organization Kochi Hospital Kochi Japan; ^4^ Department of Community Medicine for Respirology Graduate School of Biomedical Sciences, Tokushima University Tokushima Japan; ^5^ Department of Respiratory Medicine Japanese Red Cross Kochi Hospital Kochi Japan; ^6^ Department of Respiratory Medicine and Allergy Tosei General Hospital Seto Japan; ^7^ Department of Pathology National Hospital Organization Kochi Hospital Kochi Japan; ^8^ Department of Respiratory Medicine National Center for Global Health and Medicine Tokyo Japan

**Keywords:** biomarker, computed tomography, immune checkpoint inhibitors, non‐small cell lung cancer, tumor necrosis

## Abstract

**Background:**

The prognostic value of CT‐detected tumor necrosis in advanced non‐small cell lung cancer (NSCLC) treated with immune checkpoint inhibitor (ICI) monotherapy remains unclear. This two‐center retrospective study investigated whether tumor necrosis on pretreatment CT independently predicts overall survival (OS).

**Methods:**

A total of 151 advanced NSCLC patients who received ICI monotherapy between March 2016 and August 2021 at two Japanese institutions were retrospectively analyzed. Tumor necrosis was visually assessed and operationally defined as an intratumoral area of low attenuation or cavitary lesion ≥ 5 mm on pretreatment chest CT (contrast‐enhanced in 113 patients [75%] and non‐contrast in 38 [25%]). Cox regression analyses identified independent prognostic factors for OS, with internal validation using 1000 bootstrap resamples.

**Results:**

Among 151 patients (mean age 69 years; 90% with ECOG PS 0–1), 121 (80%) had CT‐detected necrosis. Median OS was significantly shorter in necrosis‐positive patients than in necrosis‐negative patients (374 vs. 1035 days; log‐rank *p* = 0.02). A multivariate Cox regression analysis identified tumor necrosis as an independent adverse prognostic factor (HR = 1.84, 95% CI = 1.03–3.30; *p* = 0.04), along with blood‐based biomarkers, tumor size, and PD‐L1 expression. The detection rate of necrosis differed between CT modalities (contrast‐enhanced 84% vs. non‐contrast 68%, *p* = 0.04).

**Conclusion:**

This exploratory two‐center retrospective study indicates the potential of CT‐detected tumor necrosis as a readily accessible prognostic marker in ICI‐treated NSCLC. However, the results obtained require validation in larger, prospective, multicenter studies with standardized CT imaging protocols before clinical implementation.

## Graphical abstract

CT‐detected tumor necrosis independently predicted worse overall survival in patients with advanced NSCLC receiving ICI monotherapy (median OS, 374 vs. 1035 days; log‐rank *p* = 0.02), supporting its potential as a simple, clinically accessible prognostic imaging biomarker.

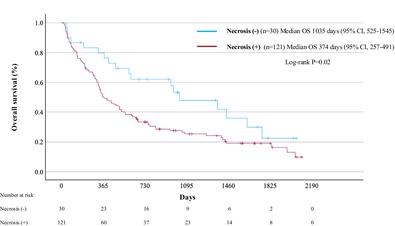

**Abbreviations**
Alb: albumin
BOR: best overall response
CI: confidence interval
CR: complete response
CRP: C‐reactive protein
CT: computed tomography
DCR: disease control rate
ECOG: Eastern Cooperative Oncology Group
Eo: eosinophils
HR: hazard ratio
ICIs: immune checkpoint inhibitors
NSCLC: non‐small cell lung cancer
ORR: objective response rate
OS: overall survival
PD: progressive disease
PD‐L1: programmed death ligand 1
PET/CT: positron emission tomography/computed tomography
PR: partial response
PS: performance status
REC: relative eosinophil count
RLC: relative lymphocyte count
RNC: relative neutrophil count
SD: stable disease
sNLR: the neutrophil‐to‐lymphocyte ratio in serum
TNM: tumor‐node‐metastasis

## 1 Introduction

Immune checkpoint inhibitors (ICIs) prolong survival in some patients with advanced non‐small cell lung cancer (NSCLC); however, long‐term outcomes remain highly heterogeneous. Simple, robust prognostic markers are needed to better stratify patients and guide treatment decisions.

Tumor necrosis is considered to reflect an aggressive tumor biology, including rapid proliferation, hypoxia, and an imbalance between tumor growth and vascular supply [1, 2, 3]. Pathological studies previously demonstrated that intratumoral necrosis was associated with a poor prognosis after the curative resection of NSCLC. In early‐stage disease, tumor necrosis has been reported as an independent risk factor for recurrence and survival in pathological stage I NSCLC [4, 5]. In stage I–IIIA NSCLC, extensive necrosis was identified as an independent adverse prognostic factor after adjustments for tumor size and clinicopathological variables [5]. A more recent study confirmed that histological necrosis effectively stratified risk in pN0 disease [6]. In addition to these findings from mixed NSCLC cohorts, a pathology‐based study focusing specifically on lung squamous cell carcinoma identified histological tumor necrosis as an independent adverse prognostic factor for disease‐free survival after curative resection [7].

Beyond histology, imaging studies have examined necrosis‐related features as radiological markers of aggressive behavior. Central low attenuation or cavitary components in computed tomography (CT)‐based assessments have been linked to poor outcomes in surgically resected NSCLC and related histological subtypes [8]. In locally advanced disease, the presence of necrosis on staging ^18^F‐fluorodeoxyglucose‐positron emission tomography/computed tomography (^18^F‐fluorodeoxyglucose‐PET/CT) has been associated with shorter progression‐free survival in patients with stage IIIB NSCLC treated with chemoradiotherapy [9].

In the era of ICIs, imaging biomarkers that predict benefits or resistance to immunotherapy are attracting increasing interest. Pretreatment pulmonary tumor necrosis on contrast‐enhanced chest CT has recently been reported as a negative prognostic imaging biomarker in advanced lung squamous cell carcinoma patients receiving first‐line anti‐programmed death 1/programmed death‐ligand 1 (PD‐L1) therapy [10, 11]. However, the majority of imaging studies on tumor necrosis have relied on contrast‐enhanced CT or PET/CT [9, 10, 11], and real‐world data using routine chest CT (with or without contrast) remain limited. Furthermore, it remains unclear whether CT‐detected necrosis independently predicts outcomes after adjusting for established prognostic factors, including blood‐based biomarkers and PD‐L1 expression, in ICI‐treated NSCLC patients.

Therefore, this two‐center retrospective study investigated whether tumor necrosis on routine pretreatment chest CT independently predicted clinical outcomes in patients with advanced or metastatic NSCLC treated with ICI monotherapy and examined its potential utility as a pragmatic imaging biomarker in real‐world clinical practice.

## 2 Materials and Methods

### 2.1 Patient Selection

**FIGURE 1:**
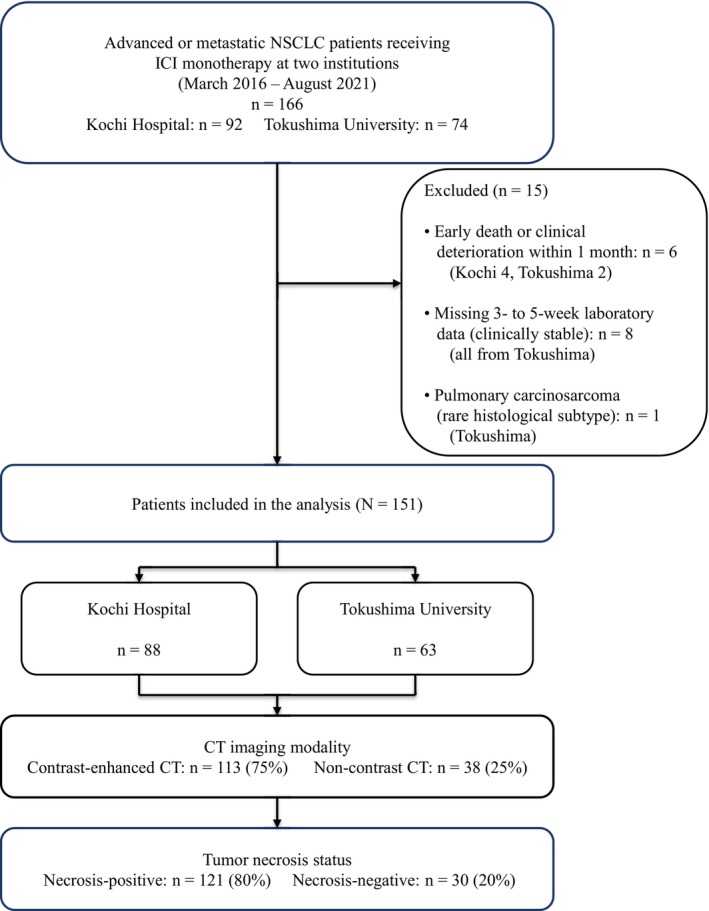
Flowchart of patient selection. Of 166 patients with advanced or metastatic NSCLC who received ICI monotherapy at two Japanese institutions between March 2016 and August 2021, 15 were excluded: 6 for early death or clinical deterioration within 1 month (Kochi 4, Tokushima 2), 8 for missing 3‐ to 5‐week follow‐up laboratory data in clinically stable patients (all from Tokushima), and 1 for pulmonary carcinosarcoma (Tokushima). The final analysis included 151 patients (Kochi 88; Tokushima 63). NSCLC = non‐small cell lung cancer; ICI = immune checkpoint inhibitor; CT = computed tomography.

This retrospective cohort study enrolled consecutive patients with advanced or metastatic NSCLC who received ICI monotherapy (nivolumab, pembrolizumab, or atezolizumab) at National Hospital Organization Kochi Hospital (*n* = 92) and Tokushima University (*n* = 74) between March 2016 and August 2021 (total *n* = 166). Fifteen patients were excluded: 6 for early death or clinical deterioration within 1 month (Kochi 4, Tokushima 2), 8 for missing 3‐ to 5‐week follow‐up laboratory data in clinically stable patients (all from Tokushima), and 1 for pulmonary carcinosarcoma (a rare histological subtype) to maintain histological homogeneity (Tokushima). The final analytic cohort comprised 151 consecutive patients (Kochi 88; Tokushima 63) for whom peripheral blood counts approximately 4 weeks after the initiation of ICIs were available (Figure 1).

### 2.2 Data Collection

Pretreatment chest CT was performed at each institution as part of routine clinical practice using multi‐detector CT scanners. Scans were acquired with or without intravenous contrast according to routine clinical indications, typically at 120 kVp with automatic tube current modulation. Axial images were reconstructed with a slice thickness of 1–2 mm using a standard (soft‐tissue) reconstruction kernel. To assess tumor necrosis, images were reviewed on mediastinal window settings, and additional lung window images were referred to as needed.

Based on previously established CT imaging criteria for pulmonary tumor necrosis [8, 11], together with related evidence regarding low attenuation necrotic components on non‐contrast CT [12], pulmonary tumor necrosis was defined as a central or eccentric area of low attenuation within the solid tumor component, including cavitary lesions, measuring ≥ 5 mm at the shortest diameter. On contrast‐enhanced CT, necrosis was identified as an area of low attenuation lacking enhancement [8, 11]. On non‐contrast CT, necrosis was assessed morphologically as a focal area of low attenuation corresponding to a necrotic component [12], including cavitary changes; however, it was not possible to evaluate the enhancement status.

In the present study, when available, necrosis was evaluated on contrast‐enhanced images; in patients who did not undergo contrast‐enhanced CT, necrosis was assessed on non‐contrast images. Since this was a retrospective multicenter study, detailed acquisition parameters, such as the contrast dose, injection rate, and scan timing, were not fully standardized or consistently retrievable for all patients. For the purposes of this study, tumor necrosis on pretreatment chest CT was operationally defined as follows: a candidate necrotic focus was required to (1) show visibly lower attenuation than the adjacent viable tumor tissue [13], (2) be free of obvious bronchial or vascular structures, and (3) not be contiguous with the tumor margin, instead appearing as an irregular or ovoid area of low attenuation within the tumor. Lesions with a maximum diameter ≥ 5 mm satisfying these criteria were classified as necrotic [10, 14], including cavitary lesions as advanced necrotic changes per the Fujisaki criteria [8]. To enhance specificity, lesions were not classified as necrotic if they showed (i) smoothly marginated homogeneous areas of low attenuation suggestive of mucinous components, (ii) hyperattenuating areas (40–70 HU) suggestive of hemorrhage, or (iii) apparent low attenuation at tumor margins attributable to partial volume effects.

Pretreatment CT images were independently reviewed by two board‐certified respiratory physicians who were blinded to clinical outcomes and to each other's assessments. Interobserver agreement for the presence of CT‐detected tumor necrosis was assessed using Cohen's kappa statistic. Cases with discrepant classifications were resolved by a consensus review.

**FIGURE 2:**
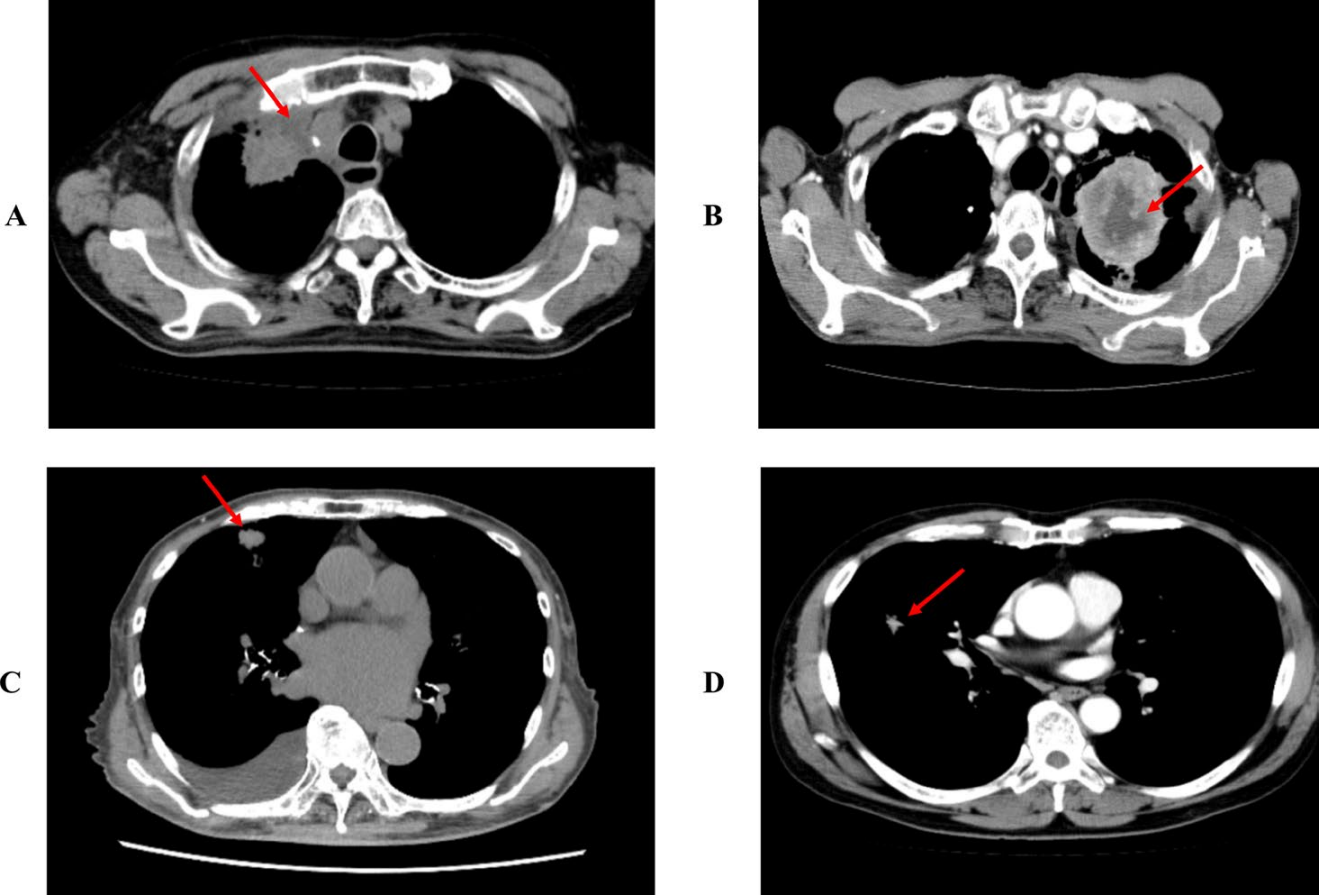
Representative pretreatment chest CT images demonstrating a tumor necrosis assessment. (A) Necrosis‐positive case on non‐contrast CT showing irregular areas of low attenuation (red arrows) within the primary tumor. (B) Necrosis‐positive case on contrast‐enhanced CT demonstrating non‐enhancing focal areas of low attenuation (red arrow) within the tumor. (C) Necrosis‐negative case on non‐contrast CT without discernible areas of low attenuation ≥ 5 mm. (D) Necrosis‐negative case on contrast‐enhanced CT. Note the difference in necrosis detectability between contrast‐enhanced (panels B, D) and non‐contrast (panels A, C) imaging modalities. CT, computed tomography.

Patients were categorized as “necrosis present” if at least one such lesion was identified within the primary tumor, and as “necrosis absent” otherwise. Representative pretreatment chest CT images are shown in Figure 2.

Baseline data included demographics (age, sex, and the smoking status), the Eastern Cooperative Oncology Group performance status (ECOG PS), laboratory indices (white blood cells [WBC], neutrophils, lymphocytes, and eosinophils [Eo]; the serum neutrophil‐to‐lymphocyte ratio [sNLR]; C‐reactive protein [CRP]; albumin [Alb]), pathology (histology and PD‐L1), ICI agent/line, start date, and vital status. Follow‐up differentials (WBC, neutrophils, lymphocytes, and Eo) were captured approximately 4 (3–5) weeks after the initiation of ICIs. Tumor metrics comprised the primary lesion size on chest CT, the number of metastatic organs, site‐specific metastases (non‐regional lymph nodes, the contralateral lung, pleura, brain, liver, kidney, adrenal gland, and bone), and staging according to the 8th edition of the TNM classification.

Responses were assessed by the Response Evaluation Criteria in Solid Tumors version 1.1 [15]; the objective response rate (ORR) = complete response (CR) + partial response (PR), and the disease control rate (DCR) = CR + PR + stable disease (SD). Overall survival (OS) was defined as the time from the initiation of ICI monotherapy to death from any cause.

### 2.3 Statistical Analysis

We used the baseline medians of CRP and Alb as cut‐off values, as previously reported [16]. Thresholds of 100/μL ≤ Eo < 500/μL, sNLR < 5, and tumor size < 5 cm were also applied [17, 18, 19]. Categorical and continuous variables were summarized descriptively. Differences in continuous variables were examined with the independent‐samples *t*‐test. Relationships among categorical variables were assessed using Pearson's *χ* ^2^ test and Fisher's exact test. OS was defined as the time from ICI initiation to death from any cause and was analyzed using the Kaplan–Meier method; survival curves were compared using the log‐rank test. Hazard ratios (HR) with 95% confidence intervals (CI) were estimated via a Cox proportional hazards model. Since the PD‐L1 status was missing in only three patients, no imputation was performed, and a complete‐case analysis was used. Internal validation was conducted with 1000 bootstrap resamples for the main multivariable model. Given the baseline difference in sNLR between the necrosis‐positive and ‐negative groups (Table 1), a sensitivity analysis was performed by including sNLR in the multivariable model to assess potential confounders. SPSS version 30.0 (IBM, Armonk, USA) was used for all analyses. Due to the exploratory nature of the study, *p* values were reported without adjustments for multiple comparisons.

**TABLE 1:** Baseline clinical characteristics of 151 patients with advanced NSCLC treated with immune checkpoint inhibitor monotherapy, stratified by the CT‐detected tumor necrosis status.

		Total	Tumor necrosis (−)	Tumor necrosis (+)	*p*
		*n* = 151	*n* = 30	*n* = 121	
CT modality, *n* (%)	Contrast‐enhanced	113 (75)	18 (60)	95 (79)	0.04^a^
	Non‐contrast	38 (25)	12 (40)	26 (21)	
Age, y	Mean (SD)	69 (9)	69 (8)	69 (9)	1.0^b^
Sex, *n* (%)	Male	117 (77)	21 (70)	96 (79)	0.3^c^
	Female	34 (23)	9 (30)	25 (21)	
Smoking history, *n* (%)	Yes	118 (78)	21 (70)	97 (80)	0.4^c^
	No	28 (19)	7 (23)	21 (17)	
ECOG PS, *n* (%)	0–1	136 (90)	28 (93)	108 (89)	0.7^c^
	2–4	15 (10)	2 (7)	13 (11)	
Stage, *n* (%)	Recurrence	21 (14)	8 (27)	13 (11)	0.04^a^
	III	30 (20)	3 (10)	27 (22)	
	IV	100 (66)	19 (63)	81 (67)	
Histological type, *n* (%)	Adeno	89 (59)	22 (73)	67 (55)	0.5^a^
	Squamous	43 (28)	6 (20)	37 (31)	
	Others	19 (13)	2 (7)	17 (14)	
PD‐L1, *n* (%)	< 1%	30 (20)	8 (27)	22 (18)	0.3^a^
	≥ 1%	118 (78)	21 (70)	97 (80)	
	Missing	3 (2)	1 (3)	2 (2)	
Treatment line, *n* (%)	1	40 (26)	4 (13)	36 (30)	0.1^c^
	> 1	111 (74)	26 (87)	85 (70)	
ICI drug, *n* (%)	Pembrolizumab	117 (77)	21 (70)	96 (79)	0.1^a^
	Nivolumab	33 (22)	8 (27)	25 (21)	
	Atezolizumab	1 (1)	1 (3)	0 (0)	
Liver metastasis, *n* (%)	No	138 (91)	28 (93)	110 (91)	1.0^c^
	Yes	13 (9)	2 (7)	11 (9)	
Brain metastasis, *n* (%)	No	117 (77)	26 (87)	91 (75)	0.2^c^
	Yes	34 (23)	4 (13)	30 (25)	
Steroids, *n* (%)	No	142 (94)	28 (93)	114 (94)	1.0^c^
	Yes	9 (6)	2 (7)	7 (6)	
Tumor size, mm	Mean (SD)	43 (23)	26 (15)	47 (23)	0.001^b^
White blood cells, /μL	Mean (SD)	6826 (2597)	6095 (2230)	7007 (2658)	0.09^b^
Neutrophils, /μL	Mean (SD)	4647 (2231)	3949 (1658)	4820 (2325)	0.06^b^
Neutrophils, %	Mean (SD)	66 (9)	64 (9)	67 (9)	0.2^b^
Lymphocytes, /μL	Mean (SD)	1440 (581)	1488 (722)	1428 (544)	0.6^b^
Lymphocytes, %	Mean (SD)	22 (9)	24 (8)	22 (9)	0.1^b^
Eosinophils, /μL	Mean (SD)	179 (187)	178 (160)	179 (193)	1.0^b^
Eosinophils, %	Mean (SD)	2.7 (2.5)	3.1 (2.8)	2.6 (2.4)	0.3^b^
sNLR, ratio	Mean (SD)	3.6 (2.0)	3.0 (1.4)	3.8 (2.1)	0.02^b^
CRP, mg/dL	Mean (SD)	2.3 (3.1)	0.8 (1.3)	2.7 (3.3)	< 0.001^b^
Alb, g/dL	Mean (SD)	3.5 (0.6)	3.8 (0.6)	3.5 (0.6)	0.03^b^
4w White blood cells, /μL	Mean (SD)	7929 (5170)	6486 (2713)	8287 (5566)	0.09^b^
4w Neutrophils, /μL	Mean (SD)	5625 (4993)	4316 (2451)	5949 (5402)	0.1^b^
4w Neutrophils, %	Mean (SD)	67 (12)	63 (11)	67 (13)	0.1^b^
4w Lymphocytes, /μL	Mean (SD)	1516 (694)	1406 (555)	1544 (724)	0.3^b^
4w Lymphocytes, %	Mean (SD)	22 (10)	24 (9)	22 (10)	0.3^b^
4w Eosinophils, /μL	Mean (SD)	218 (246)	263 (396)	207 (192)	0.5^b^
4w Eosinophils, %	Mean (SD)	3.3 (3.4)	4.3 (4.9)	3.1 (2.9)	0.2^b^
4w sNLR, ratio	Mean (SD)	5.2 (7.5)	3.7 (3.5)	5.6 (8.2)	0.2^b^

Abbreviations: Alb, albumin; CRP, C‐reactive protein; ECOG PS, Eastern Cooperative Oncology Group Performance Status; ICI, immune checkpoint inhibitor; NSCLC, non‐small cell lung cancer; PD‐L1, programmed death ligand 1; sNLR, the neutrophil‐to‐lymphocyte ratio in serum; 4w, 4 weeks later.

^a^ *χ* ^2^ test.

^b^ Independent samples *t*‐test.

^c^ Fisher's exact test.

## 3 Results

### 3.1 Patient Characteristics

A total of 151 patients with advanced or metastatic NSCLC treated with ICI monotherapy were analyzed in the present study. The patient selection process is shown in Figure 1. Table 1 summarizes the baseline clinical characteristics of these patients.

Mean age at the initiation of ICI monotherapy was 69 years. There were 117 (77%) male patients and 118 (78%) ex‐ or current smokers. Smoking history was missing for five patients. The ECOG PS of the majority of patients (90%) was 0–1. Postoperative recurrence was detected in 21 patients (14%), 30 (20%) had stage III disease, and 100 (66%) had stage IV disease. Regarding histology, 89 patients (59%) had adenocarcinoma and 43 (28%) had squamous cell carcinoma. PD‐L1 expression was positive (≥ 1%) in 118 patients (78%) and negative in 30 (20%). ICI monotherapy was the first‐line treatment in 40 patients (26%) and the second‐line or later treatment in 111 (74%). As for treatment agents, 117 patients (77%) received pembrolizumab, 33 (22%) nivolumab, and one (1%) atezolizumab. Liver metastases were detected in 13 patients (9%) and brain metastases in 34 (23%). Nine patients (6%) were receiving regular oral corticosteroid therapy during the ICI treatment. Of the 151 patients, 113 (75%) underwent pretreatment contrast‐enhanced chest CT, while the remaining 38 (25%) underwent non‐contrast CT only. Necrosis was less frequently detected on non‐contrast CT than on contrast‐enhanced CT (26/38 [68%] vs. 95/113 [84%], *p* = 0.04; Table 1).

Patients were categorized based on the presence of tumor necrosis (necrosis‐positive vs. necrosis‐negative). In the necrosis‐negative group (*n* = 30), mean age at the initiation of ICIs was 69 years, 21 patients (70%) were male, and 21 (70%) were ex‐ or current smokers. The ECOG PS of the majority of patients (28 [93%]) was 0–1. Postoperative recurrence was detected in 8 patients (27%), 3 (10%) had stage III disease, and 19 (63%) had stage IV disease. Histologically, 22 patients (73%) had adenocarcinoma and 6 (20%) had squamous cell carcinoma. PD‐L1 expression was positive in 21 patients (70%) and negative in 8 (27%). ICI monotherapy was the first‐line treatment in 4 patients (13%) and the second‐line or later treatment in 26 (87%). Liver metastases were present in 2 patients (7%) and brain metastases in 4 (13%). Two patients (7%) were receiving regular oral corticosteroid therapy during the ICI treatment.

In the necrosis‐positive group (*n* = 121), mean age at the initiation of ICI was 69 years. There were 96 male patients (79%) and 97 (80%) were ex‐ or current smokers. The ECOG PS in 108 patients (89%) was 0–1. Postoperative recurrence was detected in 13 patients (11%); 27 (22%) had stage III disease, and 81 (67%) had stage IV disease. Histologically, 67 patients (55%) had adenocarcinoma and 37 (31%) had squamous cell carcinoma. PD‐L1 expression was positive in 97 patients (80%) and negative in 22 (18%). ICI monotherapy was the first‐line treatment in 36 patients (30%) and the second‐line or later treatment in 85 (70%). Liver metastases were detected in 11 patients (9%) and brain metastases in 30 (25%). Seven patients (6%) were receiving regular oral corticosteroid therapy during the ICI treatment.

The necrosis‐positive group had a more advanced baseline stage, larger tumor size, higher sNLR and CRP levels, and lower Alb levels than the necrosis‐negative group. No significant differences were observed in WBC, neutrophil, lymphocyte, or Eo counts at baseline or 4 weeks after the initiation of ICI monotherapy between the groups.

Interobserver agreement for CT‐detected tumor necrosis was moderate, with an overall agreement of 86% and Cohen's kappa coefficient of 0.604.

### 3.2 Best Overall Response (BOR) by the Presence or Absence of Tumor Necrosis

In the overall cohort, the distribution of BOR was CR in 3/151 (2%), PR in 47/151 (31%), SD in 53/151 (35%), and progressive disease (PD) in 48/151 (32%). When stratified by tumor necrosis, BOR did not significantly differ between the groups (*p* = 0.20): necrosis‐negative (*n* = 30)—CR 1 (3%), PR 6 (20%), SD 15 (50%), and PD 8 (27%); necrosis‐positive (*n* = 121)—CR 2 (2%), PR 41 (34%), SD 38 (31%), and PD 40 (33%). Accordingly, ORR (CR + PR) was 23% in the necrosis‐negative group versus 36% in the necrosis‐positive group, while DCR (CR + PR + SD) was 73% versus 67%, respectively; neither difference was significant (Table 2).

**TABLE 2:** Best overall response by the presence or absence of tumor necrosis.

		Total	Tumor necrosis (−)	Tumor necrosis (+)	*p*
		*n* = 151	*n* = 30	*n* = 121	
Response, *n* (%)	CR	3 (2)	1 (3)	2 (2)	
	PR	47 (31)	6 (20)	41 (34)	
	SD	53 (35)	15 (50)	38 (31)	
	PD	48 (32)	8 (27)	40 (33)	0.2^a^
	ORR	50 (33)	7 (23)	43 (36)	0.3^b^
	DCR	103 (68)	22 (73)	81 (67)	0.7^b^

*Note:* ORR = CR + PR; DCR = CR + PR + SD.

Abbreviations: CR, complete response; DCR, disease control rate; ORR, objective response rate; PD, progressive disease; PR, partial response; SD, stable disease.

^a^ *χ* ^2^ test.

^b^ Fisher's exact test.

### 3.3 OS In NSCLC Patients Treated With ICIs

**FIGURE 3:**
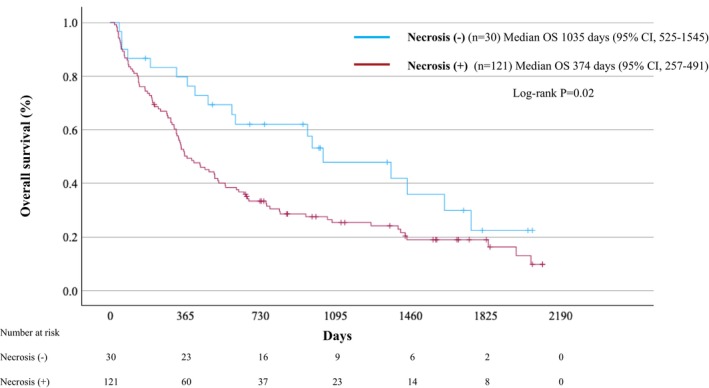
Kaplan–Meier curves for overall survival stratified by the tumor necrosis status.

In 151 NSCLC patients treated with ICIs, median OS was 476 days (95% CI, 338–614). According to the tumor necrosis status, median OS was significantly longer in the necrosis‐negative group (*n* = 30) than in the necrosis‐positive group (*n* = 121) (1035 days [95% CI, 525–1545] vs. 374 days [95% CI, 257–491], respectively; log‐rank *p* = 0.02) (Figure 3).

### 3.4 Univariate Analysis

In univariate Cox proportional hazards analyses, ECOG PS ≥ 2 (HR = 1.85, 95% CI = 1.02–3.40; *p* = 0.045), liver metastasis (HR = 1.95, 95% CI = 1.04–3.65; *p* = 0.04), 100/μL ≤ Eo < 500/μL (HR = 0.60, 95% CI = 0.41–0.89; *p* = 0.01), an elevated relative Eo count (REC) (HR = 0.49, 95% CI = 0.34–0.71; *p* < 0.001), elevated relative neutrophil count (RNC) (HR = 1.98, 95% CI = 1.07–3.65; *p* = 0.03), elevated relative lymphocyte count (RLC) (HR = 0.54, 95% CI = 0.37–0.79; *p* = 0.001), CRP ≥ 1 mg/dL (HR = 1.49, 95% CI = 1.03–2.15; *p* = 0.04), Alb ≥ 3.5 g/dL (HR = 0.68, 95% CI = 0.47–0.999; *p* = 0.049), tumor size ≥ 5 cm (HR = 1.81, 95% CI = 1.24–2.64; *p* = 0.002), and tumor necrosis (HR = 1.79, 95% CI = 1.08–2.96; *p* = 0.02) were identified as significant predictors of OS during ICI monotherapy, whereas age, sex, the disease stage, histological subtype, brain metastasis, sNLR, and PD‐L1 ≥ 1% were not (Table 3).

**TABLE 3:** A univariate Cox regression analysis of clinical and laboratory parameters associated with overall survival in patients with non‐small cell lung cancer receiving immune checkpoint inhibitors.

Parameters	Category	Hazard ratios	95% CI of HR	*p*
ECOG PS	2–4	1.85	1.02–3.40	0.045
	0–1	Reference		
Age	≥ 75	0.97	0.62–1.50	0.9
	< 75	Reference		
Sex	Female	1.18	0.77–1.82	0.5
	Male	Reference		
Stage	Recurrence	0.65	0.36–1.18	0.2
	III	0.72	0.44–1.16	0.2
	IV	Reference		
Histological type	Sq	1.15	0.75–1.66	0.6
	Non‐Sq	Reference		
Liver metastasis	Yes	1.95	1.04–3.65	0.04
	No	Reference		
Brain metastasis	Yes	1.34	0.87–2.05	0.20
	No	Reference		
Eosinophils	≥ 500/μL	2.00	0.89–4.48	0.09
	100 to < 500/μL	0.60	0.41–0.89	0.01
	< 100/μL	Reference		
Elevated REC	Yes	0.49	0.34–0.71	< 0.001
	No	Reference		
Elevated RNC	Yes	1.98	1.07–3.65	0.03
	No	Reference		
Elevated RLC	Yes	0.54	0.37–0.79	0.001
	No	Reference		
sNLR, ratio	≥ 5	0.93	0.56–1.54	0.8
	< 5	Reference		
CRP	≥ 1 mg/dL	1.49	1.03–2.15	0.04
	< 1 mg/dL	Reference		
Alb	≥ 3.5 g/dL	0.68	0.47–0.999	0.049
	< 3.5 g/dL	Reference		
Tumor size	≥ 5 cm	1.81	1.24–2.64	0.002
	< 5 cm	Reference		
PD‐L1	≥ 1%	0.67	0.43–1.03	0.07
	< 1%	Reference		
Tumor necrosis	Yes	1.79	1.08–2.96	0.02
	No	Reference		

Abbreviations: Alb, albumin; CI, confidence interval; CRP, C‐reactive protein; ECOG PS, Eastern Cooperative Oncology Group Performance Status; HR, hazard ratio; Non‐Sq, non‐squamous cell carcinoma; PD‐L1, programmed death ligand 1; REC, relative eosinophil count; RLC, relative lymphocyte count; RNC, relative neutrophil count; sNLR, the neutrophil‐to‐lymphocyte ratio in serum; Sq, squamous cell carcinoma.

### 3.5 Multivariate Analysis

Variables in the univariate model with a *p* value ≤ 0.2 were subjected to further analysis in a multivariate model. Multicollinearity among covariates was assessed using variance inflation factors; all were ≤ 2, indicating no significant multicollinearity. The Cox proportional regression analysis identified 100/μL ≤ Eo < 500/μL (HR = 0.50, 95% CI = 0.32–0.81; *p* = 0.004), an elevated REC (HR = 0.50, 95% CI = 0.33–0.77; *p* = 0.002), elevated RNC (HR = 3.00, 95% CI = 1.32–6.84; *p* = 0.009), tumor size ≥ 5 cm (HR = 1.93, 95% CI = 1.09–3.40; *p* = 0.02), PD‐L1 ≥ 1% (HR = 0.55, 95% CI = 0.34–0.90; *p* = 0.02), and tumor necrosis (HR = 1.84, 95% CI = 1.03–3.30; *p* = 0.04) as significant predictors of OS (Table 4), but not ECOG PS, stage, liver metastasis, brain metastasis, an elevated RLC, CRP, or Alb. In a sensitivity analysis including sNLR, the HR for CT‐detected tumor necrosis was essentially unchanged (HR = 1.83, 95% CI = 1.03–3.26, *p* = 0.04), while ECOG PS ≥ 2 and the presence of brain metastasis became significant (data not shown).

**TABLE 4:** A multivariate Cox regression analysis of clinical and laboratory parameters associated with overall survival in patients with non‐small cell lung cancer receiving immune checkpoint inhibitors.

Parameters	Category	Hazard ratios	95% CI of HR	*p*
ECOG PS	2–4	2.00	0.98–4.07	0.06
	0–1	Reference		
Stage	Recurrence	0.77	0.41–1.46	0.4
	III	0.71	0.39–1.26	0.2
	IV	Reference		
Liver metastasis	Yes	0.90	0.43–1.92	0.8
	No	Reference		
Brain metastasis	Yes	1.61	0.98–2.63	0.06
	No	Reference		
Eosinophils	≥ 500/μL	2.52	0.86–7.34	0.09
	100 to < 500/μL	0.50	0.32–0.81	0.004
	< 100/μL	Reference		
Elevated REC	Yes	0.50	0.33–0.77	0.002
	No	Reference		
Elevated RNC	Yes	3.00	1.32–6.84	0.009
	No	Reference		
Elevated RLC	Yes	0.83	0.52–1.31	0.4
	No	Reference		
CRP	≥ 1 mg/dL	1.28	0.73–2.23	0.4
	< 1 mg/dL	Reference		
Alb	≥ 3.5 g/dL	1.24	0.71–2.17	0.4
	< 3.5 g/dL	Reference		
Tumor size	≥ 5 cm	1.93	1.09–3.40	0.02
	< 5 cm	Reference		
PD‐L1	≥ 1%	0.55	0.34–0.90	0.02
	< 1%	Reference		
Tumor necrosis	Yes	1.84	1.03–3.30	0.04
	No	Reference		

Abbreviations: Alb, albumin; CI, confidence interval; CRP, C‐reactive protein; ECOG PS, Eastern Cooperative Oncology Group Performance Status; HR, hazard ratio; PD‐L1, programmed death ligand 1; REC, relative eosinophil count; RLC, relative lymphocyte count; RNC, relative neutrophil count.

### 3.6 Validation

#### 3.6.1 Subgroup Analysis in the Contrast‐Enhanced CT Cohort

To address a potential detection bias arising from CT modality heterogeneity, we performed a sensitivity analysis restricted to the 113 patients who underwent contrast‐enhanced CT. In this subgroup, 95 patients (84%) had CT‐detected tumor necrosis and 18 (16%) did not. A multivariate Cox regression analysis identified three independent prognostic factors: tumor necrosis (HR = 2.16, 95% CI = 1.04–4.48, *p* = 0.04), 100/μL ≤Eo < 500/μL (HR = 0.51, 95% CI = 0.31–0.83, *p* = 0.007), and an elevated REC (HR = 0.41, 95% CI = 0.26–0.65, *p* < 0.001) (Table S1). The HR was higher in the contrast‐enhanced CT cohort (HR = 2.16 vs. 1.84), suggesting the stronger prognostic effects of standardized imaging. This result supports the robustness of the prognostic impact of tumor necrosis; however, a modality‐related detection bias cannot be excluded.

The Cox proportional hazards regression model was internally validated using 1000 bootstrap resamples. The bootstrap analysis yielded similar HRs to those in the original model: 100/μL ≤ Eo < 500/μL (HR = 0.50, 95% CI = 0.27–0.85; *p* = 0.008), an elevated REC (HR = 0.50, 95% CI = 0.27–0.81; *p* = 0.002), elevated RNC (HR = 3.00, 95% CI = 1.18–9.16; *p* = 0.02), tumor size ≥ 5 cm (HR = 1.93, 95% CI = 0.94–4.27; *p* = 0.06), PD‐L1 ≥ 1% (HR = 0.55, 95% CI = 0.28–1.01; *p* = 0.04), and tumor necrosis (HR = 1.84, 95% CI = 1.01–3.89; *p* = 0.049) showed similar relationships with OS, supporting the stability and validity of the main model.

## 4 Discussion

In this retrospective cohort of 151 patients with advanced or metastatic NSCLC who were receiving ICI monotherapy, pretreatment tumor necrosis on chest CT was associated with significantly shorter OS. Necrosis remained an independent adverse prognostic factor in the multivariate analysis after adjustments for ECOG PS, the disease stage, metastatic burden, inflammatory markers, tumor size, Eo‐related indices, and PD‐L1 expression. These results indicate that CT‐visible necrosis reflects an aggressive tumor biology that is incompletely captured by conventional prognostic factors. To the best of our knowledge, few studies have evaluated the independent prognostic impact of CT‐detected tumor necrosis in advanced NSCLC treated with ICI monotherapy in a real‐world setting.

The present results align with pathological studies showing that histological necrosis independently predicted poor outcomes in resected NSCLC [4, 5, 6, 7], reflecting an aggressive, hypoxia‐driven phenotype. The present study extends these findings from resected early‐stage disease to advanced NSCLC receiving ICI monotherapy, demonstrating that CT‐defined necrosis predicts OS independent of stage and treatment modality. The robustness of these results was further supported by a sensitivity analysis restricted to the 113 patients who underwent contrast‐enhanced CT. In this homogeneous imaging cohort, tumor necrosis remained an independent adverse prognostic factor with a numerically higher HR (HR = 2.16, 95% CI = 1.04–4.48, *p* = 0.04) than that in the full cohort (HR = 1.84). This suggests that the prognostic impact of tumor necrosis is more pronounced when imaging protocols are standardized, and also that the mixed‐modality cohort may have underestimated the true effect size due to lower detection rates on non‐contrast CT (68% vs. 84%, *p* = 0.04). These results underscore the importance of contrast‐enhanced CT for a reliable necrosis assessment and support the reproducibility of our results across different analytical approaches.

Imaging studies demonstrated that necrosis‐related features predicted poor outcomes in various cancers [8, 9, 10, 11]. The present results corroborate these findings, showing that necrosis on routine chest CT reflects an aggressive tumor biology. Although tumor necrosis was associated with a larger tumor size and elevated inflammatory markers (Table 1), it remained an independent predictor of worse OS (HR = 1.84, 95% CI = 1.03–3.30; *p* = 0.04; Table 4) after adjustments for these factors. An assessment of multicollinearity confirmed statistical independence (variance inflation factors ≤ 2), indicating that necrosis provides prognostic information beyond tumor burden and systemic inflammation. The high prevalence of CT‐detected necrosis (80%) in our cohort may reflect the predominance of advanced‐stage tumors with large tumor burdens in this real‐world population. Importantly, tumor necrosis remained independently associated with OS after adjustments for tumor size and systemic inflammatory markers, suggesting that it captures biological aggressiveness beyond tumor volume alone.

An early radiological response did not fully explain the survival difference. ORR and DCR did not significantly differ with the necrosis status, suggesting that necrosis relates more to the durability of disease control than the early tumor response. The paradoxical result showing that ORR was numerically higher in the necrosis‐positive group (36% vs. 23%, *p* > 0.05) despite significantly worse OS warrants mechanistic investigation. Several mechanisms may explain this dissociation. This dissociation underscores that the radiological response and underlying biological tumor aggressiveness represent distinct dimensions of the treatment outcome in the immunotherapy era. Necrosis‐associated hypoxia promotes immunosuppressive infiltration, impairs T‐cell function, and contributes to resistance through hypoxia‐inducible factor‐1α‐mediated pathways [20, 21, 22, 23]. Preclinical studies showed that targeting hypoxia may restore anti‐tumor immunity and enhance checkpoint blockade efficacy [22, 23]. Additionally, necrotic tumors with a rapid turnover may show a transient volume reduction due to necrosis itself rather than immune‐mediated tumor control. These findings underscore that imaging biomarkers require evaluation beyond the early response, encompassing the durability of control.

The mechanisms linking tumor necrosis to poor ICI outcomes may involve both local and systemic factors. Necrosis reflects rapid proliferation outstripping vascular supply, resulting in hypoxia. The hypoxic microenvironment promotes immune evasion through hypoxia‐inducible factor‐1α signaling, vascular endothelial growth factor production, and the accumulation of immunosuppressive cells, collectively impairing T‐cell infiltration and function [20, 21, 22]. Additionally, necrotic areas exhibit poor perfusion, potentially limiting antibody delivery. Highly necrotic tumors may harbor aggressive clones with enhanced metastatic potential. These factors may diminish ICI efficacy despite similar initial responses.

Clinically, pretreatment chest CT provides readily interpretable prognostic information for NSCLC patients receiving ICI monotherapy. The detection of tumor necrosis may help identify high‐risk patients despite immunotherapy, prompting closer monitoring and alternative treatment strategies. Patients with necrosis may preferentially receive ICI‐chemotherapy combinations, ICI plus anti‐angiogenic therapy, or novel combination regimens in clinical trials. In contrast, ICI monotherapy may suit patients without necrosis and favorable features. Additionally, the necrosis status may be incorporated into multivariable prognostic models alongside PD‐L1 expression, tumor size, and blood‐based markers that also predicted survival in our cohort.

This study has several limitations that need to be addressed. This was a small, two‐center retrospective study (*N* = 151), which limits its statistical power, precludes causal inference, and may reduce generalizability. Furthermore, CT imaging was heterogeneous (contrast‐enhanced and non‐contrast), which may have introduced a detection bias. However, this heterogeneity reflects real‐world clinical practice. Importantly, a sensitivity analysis restricted to the contrast‐enhanced CT subgroup confirmed the independent prognostic impact of tumor necrosis, supporting the robustness of our results. Although interobserver reproducibility for CT‐detected tumor necrosis was formally evaluated and showed moderate agreement (overall agreement, 86%; Cohen's kappa = 0.604), some degree of subjectivity in a visual qualitative assessment remains unavoidable. In addition, a necrosis assessment was performed using both contrast‐enhanced and non‐contrast CT, which are not equivalent for evaluating non‐enhancing components. Therefore, a modality‐related misclassification bias cannot be excluded. Future prospective studies using standardized contrast‐enhanced CT protocols and quantitative volumetric or radiomic analyses are warranted to further enhance methodological rigor. Furthermore, 15 patients were excluded for prognosis‐unrelated reasons (mainly missing follow‐up laboratory data) or histological heterogeneity (one pulmonary carcinosarcoma); exclusion rates for early death or clinical deterioration were similar between institutions, minimizing concerns about a survivor bias; however, a minor effect cannot be entirely excluded. Another limitation was the marked group imbalance (necrosis‐positive *n* = 121 vs. necrosis‐negative *n* = 30), which may have limited statistical precision, as reflected in the wide CI for tumor necrosis (HR = 1.84, 95% CI = 1.03–3.30). Although internal bootstrap validation with 1000 resamples supported model stability, external validation in independent cohorts is crucial for confirming generalizability before clinical implementation. Moreover, this cohort partially overlapped with our previous studies [16, 17, 24]; however, the present analysis focused on pretreatment tumor necrosis as a prognostic marker.

Despite these limitations, this study has notable strengths. We examined a clinically relevant population receiving ICI monotherapy in real‐world practice. The multivariable analysis was adjusted for comprehensive confounders, including clinical, laboratory, and imaging covariates. Additionally, by demonstrating that a simple visual necrosis assessment on routine CT independently predicts OS, the present results support its potential utility as a pragmatic, accessible imaging biomarker.

The readily accessible nature of a CT‐based necrosis assessment—requiring no additional imaging modalities or costly biomarker testing—makes it an attractive pragmatic tool for risk stratification. The detection of tumor necrosis on pretreatment CT may help identify high‐risk patients who may benefit from closer monitoring, alternative treatment strategies, or enrolment in clinical trials evaluating combination therapies targeting the hypoxic tumor microenvironment [23]. However, given the imaging heterogeneity and lack of external validation in this study, these results need to be regarded as hypothesis‐generating rather than practice‐changing. In future large‐scale prospective studies, restricting enrolment to patients who undergo standardized contrast‐enhanced CT may reduce the detection bias and improve imaging homogeneity. In addition, a quantitative CT‐based volumetric analysis, including necrotic volume and the ratio of necrotic to total tumor volume, may provide a more objective assessment of tumor necrosis and further refine its prognostic value. Large‐scale multicenter validation studies are warranted.

In conclusion, this exploratory two‐center retrospective study suggests that CT‐detected tumor necrosis on routine pretreatment chest CT is independently associated with worse OS in ICI‐treated NSCLC. Given the study's inherent limitations, large‐scale, prospective, multicenter validation is warranted before clinical implementation.

## Author Contributions

**Eiji Takeuchi:** conceptualization, data curation, formal analysis, writing – original draft, visualization. **Hirokazu Ogino:** data curation, writing – review and editing. **Kensuke Kondo:** data curation. **Kaori Nii:** data curation. **Michihiro Kunishige:** data curation. **Yugo Matsumura:** data curation. **Yoshio Okano:** data curation. **Hisanori Machida:** data curation. **Seidai Sato:** data curation, supervision. **Takuya Ishida:** data curation. **Naoki Kadota:** data curation. **Seiya Ichihara:** data curation. **Hiroshi Nokihara:** conceptualization, writing – review and editing. **Nobuo Hatakeyama:** data curation. **Keishi Naruse:** data curation. **Tsutomu Shinohara:** supervision. **Yasuhiko Nishioka:** conceptualization, writing – review and editing. **Hiroki Takahashi:** data curation.

## Funding

The authors have nothing to report.

## Ethics Statement

The study protocol received approval from the Institutional Review Boards of the National Hospital Organization Kochi Hospital (R3‐3) and Tokushima University (3013–3). Due to the retrospective design, the requirement for informed consent was waived. The study was conducted in accordance with the Declaration of Helsinki.

## Conflicts of Interest

Hirokazu Ogino reports research grant funding to his institution from Taiho Pharmaceutical. Hiroshi Nokihara reports honoraria for lectures from MSD, AstraZeneca, and Chugai Pharmaceutical. Yasuhiko Nishioka reports honoraria for lectures from Boehringer Ingelheim, AstraZeneca, and Chugai Pharmaceutical; research grant funding to his institution from TMS, SANSHO, and Taiho Pharmaceutical; and scholarship donations from Chugai Pharmaceutical, Taiho Pharmaceutical, Asahi Kasei Pharma, and Eli Lilly Japan. The remaining authors declare that this research was conducted in the absence of any commercial or financial relationships that may be construed as a potential conflicts of interest.

## Supporting information


**Table S1:** A multivariate Cox regression analysis of prognostic factors for overall survival in the contrast‐enhanced CT subgroup (*n* = 113).

## Data Availability

Raw data supporting the conclusions of this article will be made available by the authors upon reasonable request, subject to Institutional Review Board approval and data protection regulations.
