# Supplementary material for: Tumor Necrosis on Routine Pretreatment CT as a Potential Prognostic Marker in Advanced NSCLC Treated With Immune Checkpoint Inhibitor Monotherapy: A Two‐Center Retrospective Study
Source: Thorac Cancer. 2026 Jul 26;17(14):e70363. doi: 10.1111/1759-7714.70363 (PMC13401793; doi:10.1111/1759-7714.70363)
Supplement: Supplementary file 1 — Table S1: A multivariate Cox regression analysis of prognostic factors for overall survival in the contrast‐enhanced CT subgroup (n = 113). [file TCA-17-e70363-s001.docx]

**Supplementary Table S1** A multivariate Cox regression analysis of prognostic factors for overall survival in the contrast-enhanced CT subgroup (n = 113)

This sensitivity analysis was restricted to patients who underwent contrast-enhanced CT to address a potential detection bias from imaging modality heterogeneity.

| Parameters | Category | HRs | 95% CI of HRs | P value |
| --- | --- | --- | --- | --- |
| ECOG PS | 2~4 | 1.53 | 0.69-3.41 | 0.3 |
|  | 0~1 | Reference |  |  |
| Eosinophils | ≥500/μL | 1.16 | 0.38-3.56 | 0.8 |
|  | 100–<500/μL | 0.51 | 0.31-0.83 | 0.007 |
|  | <100/μL | Reference |  |  |
| Elevated REC | Yes | 0.41 | 0.26-0.65 | <0.01 |
|  | No | Reference |  |  |
| Tumor size | ≥5 cm | 1.38 | 0.86-2.24 | 0.20 |
|  | <5 cm | Reference |  |  |
| Tumor necrosis | Yes | 2.16 | 1.04-4.48 | 0.04 |
|  | No | Reference |  |  |

**Abbreviations:** HR, hazard ratio; CI, confidence interval; ECOG PS, Eastern Cooperative Oncology Group Performance Status; REC, relative eosinophil count
